# Evaluation of MR elastography as a response parameter for transarterial chemoembolization of colorectal liver metastases

**DOI:** 10.1007/s00330-020-06706-y

**Published:** 2020-02-21

**Authors:** Thomas J. Vogl, Simon S. Martin, Addison A. Johnson, Yannick Haas

**Affiliations:** 1 Department of Diagnostic and Interventional Radiology, University Hospital Frankfurt, Frankfurt, Germany; 2 Department of Radiology and Radiological Science, Medical University of South Carolina, Charleston, SC USA

**Keywords:** Liver, Chemotherapy, Metastasis, Therapeutic chemoembolization, Magnetic resonance imaging

## Abstract

**Objective:**

The aim of this study was to evaluate magnetic resonance elastography (MRE) as a response parameter in patients who received transarterial chemoembolization (TACE) for the treatment of colorectal liver metastases.

**Materials and methods:**

Forty-two patients (29 male patients; mean age, 61.5 years; range, 41–84 years) with repeated TACE therapy of colorectal liver metastases underwent on average 2 repetitive magnetic resonance imaging (MRI) and MRE exams in 4- to 6-week intervals using a 1.5-T scanner. MRE-based liver stiffness measurements were performed in normal liver parenchyma and in metastatic lesions. Moreover, the size of the liver metastases was assessed during treatment and compared with the results of the MRE analysis.

**Results:**

Liver metastases showed a significantly higher degree of stiffness compared with the normal liver parenchyma (*p* < 0.001). However, only a weak correlation was found between the lesion size and stiffness (*r* = − 0.32, *p* = 0.1). MRE analysis revealed an increase in stiffness of the colorectal liver metastases from 4.4 to 7.1 kPa after three cycles of TACE (*p* < 0.001). Also, the mean size of the metastases decreased from 17.0 to 11.3 cm^2^ (*p* < 0.001). Finally, the entire liver stiffness increased from 2.9 to 3.1 kPa over the three cycles of TACE therapy.

**Conclusion:**

In conclusion, MRE showed a significant change in stiffness and size of liver metastases. Therefore, MRE may provide an added value for an evaluation of treatment response in patients with colorectal liver metastases undergoing TACE.

**Key Points:**

*• MRE showed an increase in stiffness of the colorectal liver metastases during TACE therapy.*

*• Liver metastases showed a significantly higher degree of stiffness compared with the normal liver parenchyma.*

*• However, only a weak correlation was found between the lesion size and stiffness.*

## Introduction

Colorectal cancer is one of the most prevalent forms of cancer in the world [1], and the liver represents the most common site of metastases [2]. Overall, 50% of patients with colorectal carcinoma develop liver metastases and 20% of them have synchronous metastases, which generally show poor outcomes [3–6]. Partial liver resection can only be considered in patients with good liver function to avoid potential liver decompensation [7–9]. Systemic chemotherapy also has limited use for patients with unresectable liver metastases [8, 10–12]. Therefore, alternative therapies like RFA (radiofrequency ablation) and MWA (microwave ablation) are recommended for those patients [13–16].

If neither surgery nor local-ablative methods show response, transarterial chemoembolization (TACE) is an option for local tumor control. This therapy has shown promising results in patients with unresectable colorectal liver metastases [13]. TACE is based on the administration of a cytostatic substance directly into the tumor feeding vessel and shows fewer side effects compared with systematic chemotherapy. Due to the combination of direct chemotherapeutic application and vascular occlusion technique, the cytostatic substances are in contact with the targeted malignant cells for a longer period of time [17, 18]. Magnetic resonance imaging (MRI) and computed tomography (CT) are frequently used for therapy assessment and follow-up. Magnetic resonance elastography (MRE) is a novel technique to be considered and can be applied to most patients undergoing MRI [19, 20]. MRE showed a higher accuracy and a better reproducibility compared with sonography-based elastographic measurements [21].

The aim of our study was to assess MRE as a response parameter in patients with TACE of colorectal liver metastases.

## Material and methods

### Study design and population

This retrospective study was approved by the institutional ethical committee of our university hospital. Data was collected between April 2017 and December 2017. All patients had met our inclusion criteria as follows: (a) age between 18 and 90 years old, (b) histopathological and/or radiological evidence of colorectal liver metastases, (c) current therapy with TACE, (d) no contraindications for MRI scanning. Exclusion criteria were as follows: (a) liver metastases of different origin, (b) inflammation in or around the liver, (c) abbreviations from the standard imaging protocol.

Forty-two patients (29 men and 13 women; mean age, 61.5 years; range, 41–84 years) who had met the general inclusion and exclusion criteria were analyzed in the present study. The patients underwent regular treatment with TACE (mitomycin, gemcitabine, and lipiodol) and MRI with additional MRE. The MRI was performed either on the same day as the TACE therapy or in the following 2 days after the intervention, so that a range of 0–2 days in between MRE and TACE can be given. As treatment effects will vary over time and inconsistencies in timing between MRE snapshots and TACE could bias the results, patients with longer ranges were not included. The majority of MRI and MRE examinations (93%) were performed immediately after TACE on the same day. All study patients underwent TACE in 4–6 weeks intervals. Twelve patients had evidence of metastases in the left liver lobe, while the other 30 showed metastases in the right lobe. In the case of multiple metastatic lesions in a single patient, only the largest lesion was evaluated. On average, 3.3 (range, 0–23) TACE interventions were performed before the first MRE imaging.

### TACE interventions

After the puncture of a femoral artery, a pigtail catheter was inserted via the Seldinger technique [22]. Thereafter, a cobra or sidewinder catheter was placed in the celiac and superior mesenteric artery to visualize the vessels and liver metastases. In addition, the information from the MRI was used to identify the segmental, tumor-feeding artery using micro-catheters (size, 2.3–3.0 F) to prevent vasospasms. After positioning the catheters, the cytostatic substances were administered (mitomycin C (8 mg/m^2^ body surface), gemcitabine (500 mg/m^2^ body surface), and cisplatin (30 mg/m^2^ body surface) in all patients. After administration of the chemotherapeutic substances, a selective injection of lipiodol was performed. The lipiodol capitation was not correlated with the efficacy of the TACE because of the frequently inhomogeneous appearance. All TACE therapies were performed using a robot-supported angiography system (Artis pheno, Siemens Healthineers), and the median time of the whole procedure was 35 min including the installation of the coil and positioning checks [22].

### MRI and MRE acquisition

Unenhanced and contrast-enhanced MRI was performed directly before or after the intervention using gadobutrol (Gadovist 1 mmol/ml, Bayer Healthcare). T1- and T2-weighted MRI and MRE scans were acquired in transverse and sagittal orientation with 5 mm slice thickness using a 1.5-T system (MAGNETOM Avanto, Siemens). Commercially available system was used for MRE measurements (Resoundant) which consisted of an active driver located outside the scan room connected to passive actuators in the scan room. The time of vibrations was between 15 s for five slices of EPI (WIP measurement) and 23 s for a single slice of GRE. The median time for the MRE measurement including patient and hardware preparation was 18 min. The applied sequence protocol consisted of the listed parameters in Table 1. All images were acquired during inspiratory breath hold and with a vibration frequency of 60 Hz. The system evaluated magnitude image, phase image, wave image, color-coded elastogram, and confidence map. The stiffness metric was read in the magnitude of the complex shear modulus which could be read directly from the scanner outputs.

**Table 1:** MRE protocol

MR parameter	Value
Repetition time (TR)	50 ms
Echo time (TE)	21.1 ms
Bandwidth	250 Hz/pixel
Field of view (FOV)	400 × 400
Matrix	128 × 128
NEX	1
Slice thickness	10 mm

### MRE measurements

**Fig. 1:**
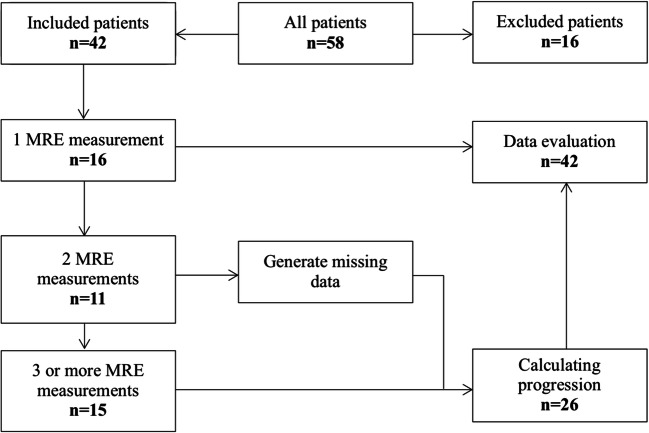
Flowchart of the study population. There were three groups of patients. Patients with one MRE imaging (*n* = 16) were only used for general elastography data collection. The patients with two (*n* = 11) or more (*n* = 15) MRE series were further compared for size and stiffness evaluation during therapy

**Fig. 2:**
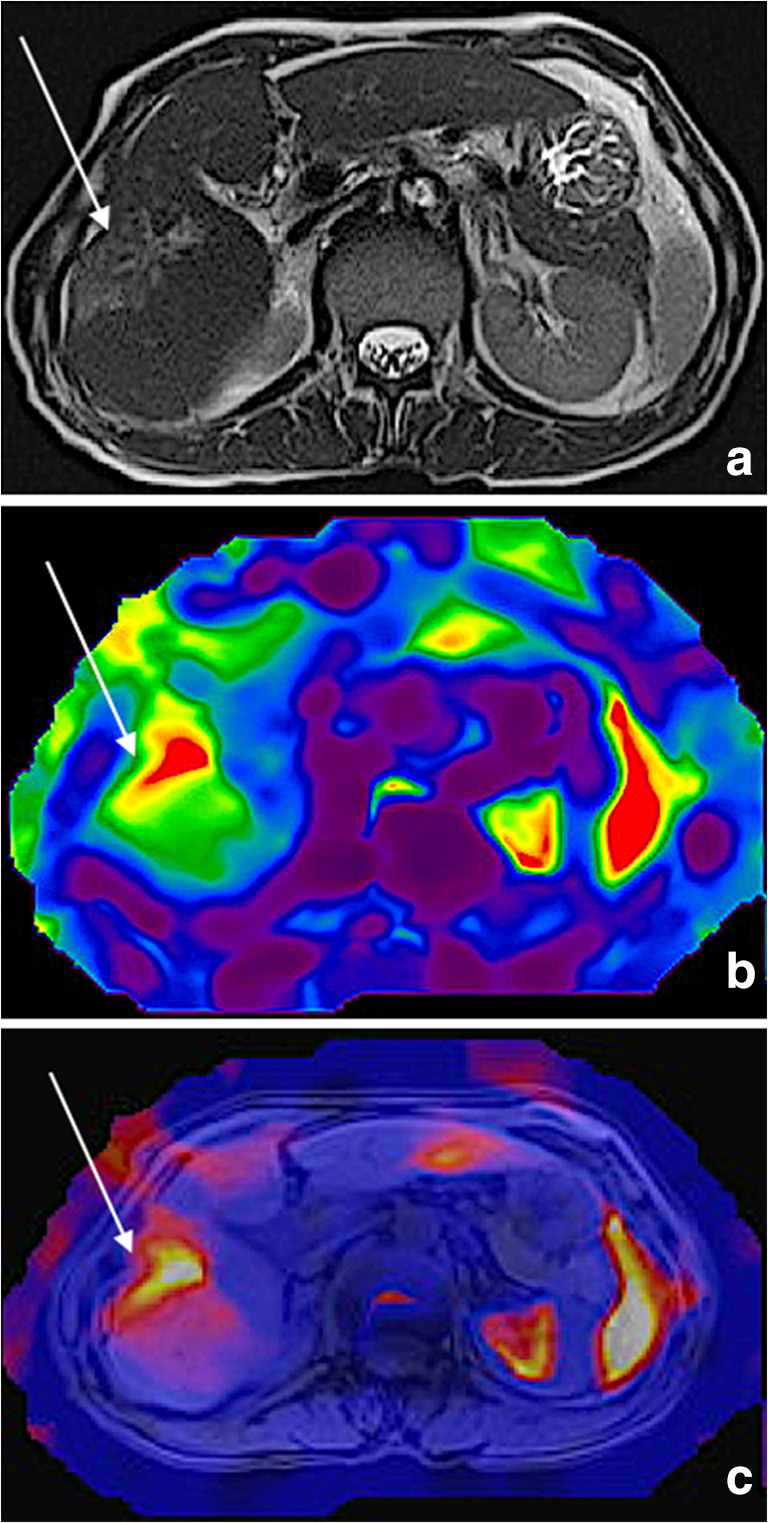
A 64-old-male patient with colorectal liver metastasis in the right hepatic lobe. The metastasis is visualized in both images (arrows). The native MRI series (**a**) were matched with MRE data and merged to a tumor-stiffness 3D fusion for parameter analysis (**b**, **c**)

**Fig. 3:**
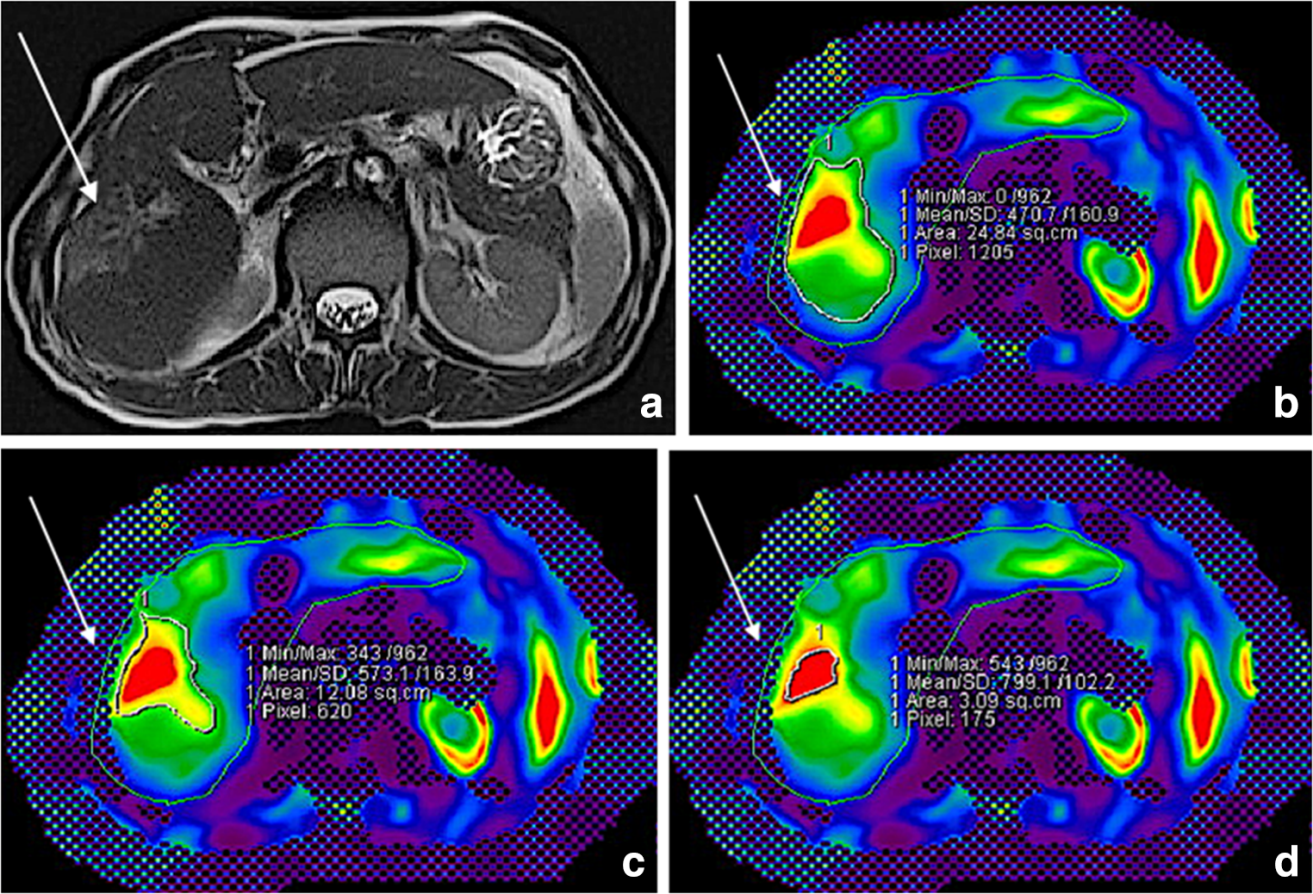
A 64-old-male patient with colorectal liver metastasis in the right hepatic lobe (arrow) and TACE therapy. Evaluation of the treatment response included the following parameters: total liver extent (cm^2^), total liver stiffness (kPa), total liver MAP T1 (ms) and MAP T2 (ms), left and right lobe separated extent, stiffness and MAP T1/T2 measurement, metastasis extent, stiffness and MAP T1/T2. The images show the T1 image (**a**), as well as MRE measurements of the surrounding area (**b** with 4.7 kPa, **c** with 5.7 kPa) and the metastasis itself (**d** with 8.0 kPa)

The MRI data was matched with the MRE measurements using a dedicated software (MapIt Software, Siemens). This approach allows for a high-resolution segregation of intrahepatic structures and exact measurements of intrahepatic elastography, differentiating metastases and healthy tissue. Three different groups of patients with MRE imaging were investigated, as not every patient had the same number of performed MRE series due to the retrospective design of the study. Patients with one MRE examination (*n* = 16) were only used for the collection of the general elastography data. Patients with two (*n* = 11) or more (*n* = 15) MRE series were evaluated to assess therapy response (Fig. 1). A third data was calculated for patients with only two measurements via linear extrapolation, as more than one cycle is necessary for this evaluation. For this study, the MRI series were matched with MRE and merged to a tumor-stiffness 3D fusion image (Fig. 2). Evaluation of the treatment response included the following parameters: total extent of the liver (cm^2^), total liver stiffness (kPa) (including healthy parenchyma and tumor), total liver MAP T1 (ms) and MAP T2 (ms), left and right lobe separated extent, stiffness and MAP T1/T2 measurement, metastasis extent, stiffness and MAP T1/T2 (Fig. 3). Additionally, measurements in the healthy liver tissue were performed for comparison.

### Statistical analysis

Minimum and maximum values with standard deviations were calculated for continuous variables, whereas medians with ranges were calculated for categorical variables. The Kolmogorov-Smirnov test was applied to test for normal distribution. The Friedman test was used to determine whether differences between the measurements were significant. Statistical software (IBM SPSS Statistics, version 25, IBM) was used and a *p* value < 0.05 indicated statistical significance. Spearman’s test was used to assess correlation.

## Results

The mean size of the metastases decreased from 15.9 ± 11.3 cm^2^ in the first MRI to 12.5 ± 8.0 cm^2^ in the second MRI and to 11.5 ± 10.0 cm^2^ in the third MRI (*p* = 0.03). Simultaneously, a significant change in stiffness was detected in the liver metastases increasing from 4.8 ± 1.4 kPa in the first MRI to 7.0 ± 2.6 kPa in the third MRI (*p* < 0.001). In addition, the metastases (5.9 ± 2.2 kPa) were significantly stiffer than the normal liver parenchyma (3.2 ± 1.0 kPa) (*p* < 0.001) (Table 2).

**Table 2:** Stiffness of different liver parts

Measurement	Cycle 1	Cycle 2	Cycle 3	Total	*p* value	SD
Area metastasis (cm^2^)	15.9	12.5	11.5	13.3	0.03	9.9
Stiffness metastasis (kPa)	4.8	5.9	5.9	5.9	< 0.001	2.2
Stiffness liver parenchyma (kPa)	3.0	3.2	3.3	3.2	> 0.05	1.0

However, only a weak correlation was found between size and stiffness measurements (*r* = − 0.32, *p* = 0.1).

**Fig. 4:**
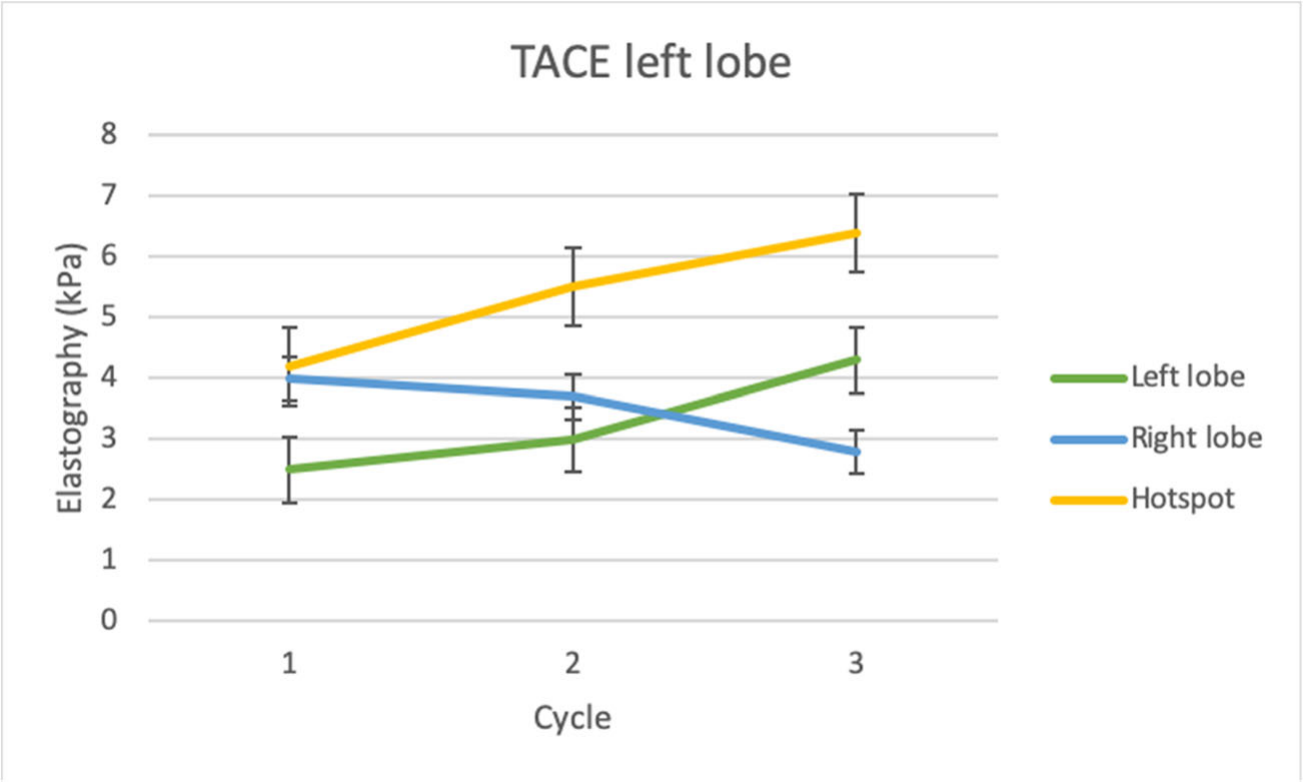
TACE and MRE measurements in patients with colorectal liver carcinoma in the left liver lobe. A significant higher increase of stiffness (+ 72%, *p* = 0.04) was observed in the left lobe compared with the right lobe (− 37.5%, *p* > 0.05) during the therapy. Additionally, the stiffness of the metastases was showing a distinct increase of stiffness (52.4%, *p* = 0.03)

In the group of patients with metastases in the left liver lobe, a considerably higher increase in stiffness parameters was detected in the left lobe (+ 72%, *p* = 0.04) compared with the right lobe (− 37.5%, *p* > 0.05) during the three cycles of TACE therapy. The left lobe parenchyma showed a significantly lower stiffness (3.3 ± 1.3 kPa) compared with the metastases (5.4 ± 1.8 kPa) (Fig. 4). The stiffness of the right lobe decreased from 4.0 ± 1.6 to 2.8 ± 2.0 kPa (*p* = 0.1) during therapy (Table 3).

**Table 3:** Stiffness of liver at left-sided treated patients

Measurement	Cycle 1	Cycle 2	Cycle 3	Total	*p* value	SD
Area metastasis (kPa)	2.5	3.0	4.3	3.3	0.04	1.3
Stiffness metastasis (kPa)	4.0	3.7	2.8	3.5	> 0.05	1.9
Stiffness liver parenchyma (kPa)	4.2	5.5	6.4	5.4	0.03	1.8

The stiffness of the liver as a whole slightly increased from 3.0 ± 0.9 kPa in the first cycle to 3.3 ± 1.2 kPa in the last cycle (*p* > 0.05). Measurements in the healthy liver tissue showed an increase in stiffness of only + 0.87% with an average of 2.2 ± 0.6 kPa during therapy (*p* > 0.05) (Table 4).

**Table 4:** Stiffness of different liver areas

Measurement	Cycle 1	Cycle 2	Cycle 3	Total	*p* value	SD
Healthy parenchyma	2.2	2.2	2.2	2.2	> 0.05	0.6
MAP T1 parenchyma (ms)	636	678	655.2	656.4	> 0.05	217
MAP T2 parenchyma (ms)	64.8	70.4	65	66.7	> 0.05	21.4
MAP T1 metastasis (ms)	766.7	815.5	790.7	791	> 0.05	307.1
MAP T2 metastasis (ms)	78.3	86.2	76	80.2	0.02	37.1

The MAP measurements showed fewer changes. The overall MAP T1 of the whole liver increased by 0.03% (from 636 ± 226.7 to 655.2 ± 236.4 ms; *p* > 0.05) and the MAP T2 of the whole liver increased by only 0.003% (from 64.8 ± 23.0 up to 65.0 ± 21.8 ms; *p* > 0.05). The MAP T1 and MAP T2 measurements of the metastases showed similar low changes than the rest of the liver. The MAP T2 in the metastases showed a significantly decrease (from 78.3 ± 38.2 to 76 ± 38.1 ms; *p* = 0.02) (Table 4).

## Discussion

The aim of this study was to evaluate the therapeutic response of TACE in patients with colorectal liver metastases using MRI and MRE. We found that the additional use of elastography in the routine MRI protocol allows for the assessment of liver stiffness during interventional TACE cycles. MRE revealed significant differences in stiffness parameters for both normal liver lobes and metastases. This trend is due to the targeted accumulation of the drugs in the affected liver lobe without diffusion to the other lobe. Furthermore, a significant decrease in size and an increase in stiffness of the metastatic lesions demonstrated the beneficial therapeutic effects of TACE therapy. Although we have only found a weak correlation, the power of their validity should not be underestimated. For this reason, larger studies should be carried out to validate our results. The comparison of both liver lobes showed a lesser increase in stiffness in the untouched liver lobe compared with the treated lobe. Therefore, MRE can be used to evaluate the tumor response of colorectal liver metastases to chemotherapeutic substances. Furthermore, many patients undergoing TACE therapy have MRI or CT for staging and follow-up examinations, what insures a lot of data without additional imaging.

Gordic et al reported a different effect in their study in patients with hepatocellular carcinoma (HCC) who had undergone yttrium-90 radioembolization, TACE, or radiofrequency ablation (RFA) [23]. In their study, a significant lower stiffness was measured in treated tumors compared with untreated tumors. Even they showed a significantly higher accumulation of drugs in the tumor compared with the rest of the liver. A lower stiffness in HCC after therapy and a higher stiffness in CRLM after TACE might be explained by the different tumor entity. Moreover, the combination of different therapies in the study of Gordic et al compared with the single use of TACE in our study may have influenced the results. Venkatesh et al [24] investigated the stiffness of liver tumors with MRE. The authors demonstrated a non-significant linear correlation between the tumor size and stiffness. In contrast to our study, no homogeneous tumor was measured, but a large range of different lesions such as metastatic lesions (14, different primary tumor), hepatocellular carcinomas (12), hemangiomas (9), cholangiocarcinomas (5), focal nodular hyperplasia (3), and hepatic adenoma (1). Due to this big difference of tumor ethnicity, the validity of the results is not as powerful as in our study with only one ethnicity. Moreover, the tumors were measured once and not in several phases. In another study, a correlation between tumor size and stiffness was also reported by Hennedige et al [25] as they demonstrated that benign and malignant lesions can be differentiated more significantly with MRE than with DWI.

Moreover, few studies evaluated the effects chemotherapy on liver stiffness in animal experiments. Pepin et al compared the stiffness of treated and untreated tumors in a mouse model [26]. After subcutaneous injection of non-Hodgkin’s lymphoma cells in mice and subsequent treatment with a chemotherapeutic agent or saline, the authors detected a decrease of stiffness after 4 days in the tumors treated with chemotherapy and no appreciable change in the tumors treated with saline. Although there was no significant volume change detectable in this short time, the authors found a decreased level of cell proliferation [26]. In comparison with our study, neither the tumor entity nor the therapy was identical. Li et al demonstrated a similar decrease of stiffness in treated tumor cells compared with the non-treated cells in human colorectal cancer xenografts before and after treatment with vascular disrupting agent ZD6126 (N-acetylcolchinol-O-phosphate). The MRE took place 24 h after the treatment. In their analysis, a central necrosis was histologically confirmed [27].

These studies all demonstrate that MRE can be used for early response evaluation of tumor cells under therapy with chemotherapeutic agents. The studies showed in general a decrease of stiffness which is contrary to our results. This might be attributable to the different tumor origin and therapy in our study. Moreover, the accumulation of lipiodol in the metastases could have modified the stiffness of the lesions and may explain the different results compared with those in the literature.

Our results showed a higher degree of stiffness in metastases than in healthy liver areas measured. However, we found only a weak correlation between stiffness and size measurements of liver metastases. To our knowledge, no previous studies have analyzed the stiffness of colorectal liver metastases using MRI and MRE. Moreover, the difference between the left lobe and right liver lobe may be explained by the method, as the vibration source is located on the right anterior chest wall with intercostal approach. In this area, the compression might be less effective than in the left lobe which can be compressed by the elastic band that maintains the system. However, almost no drug-related effects were found in the healthy liver tissue.

MRE allows for highly accurate analysis of specific liver structures. Although ultrasound elastography has a higher plane resolution than MR, the depth is very limited [18]. The acquisition of EPI data took about 15 to 23 s for 1–5 slices (WIP measurement), so a MRE measurement of the liver took on average up to 30 min. Once the software is available, elastography measurements can be performed on every 1.5-T MR scanner with the use of the dedicated hardware and software packages.

There were certain limitations in our study. First, the patient cohort was relatively small, and we did not include a control group. Second, patients with different tumor stages were included in this study. The impact of TACE varies according to different tumor stages, as well as the measured stiffness. Third, patients with MRE were treated during different phases of TACE therapy. Some patients included in the current study received their first cycle while others had more than 20 therapies completed. Fourth, there were different periods of time between the start of therapy and image acquisition that may affect our data. As our study was in a retrospective setting and many patients received their therapy for a longer period of time, it would be interesting to evaluate MRE-based parameters in a prospective study with more patients. Furthermore, some of the MRE data were extrapolated that might also create bias [28]. In general, the quality of extrapolation is limited by the assumptions about the process of MRE values during therapy. Moreover, it remains unclear how accurate and repeatable MRE examinations are, even if the examination conditions are kept identically [28]. Therefore, studies with larger patient cohorts need to be conducted to confirm our results.

In conclusion, the MRI and MRE are useful tools to evaluate tumor response after TACE therapy in patients with colorectal liver metastases. Our results showed a significant increase in stiffness of the metastases and a simultaneous decrease in size of the metastases. Therefore, MRE imaging may provide additional value to evaluation of tumor response after TACE therapy. In addition, MRE measurements may be used to calculate the remaining healthy liver proportion and function.

## Abbreviations

CRLM: Colorectal liver metastases
CT: Computed tomography
HCC: Hepatocellular carcinoma
kPa: Kilo pascal
MRE: Magnetic resonance elastography
MRI: Magnetic resonance imaging
RFA: Radiofrequency ablation
TACE: Transarterial chemoembolization
